# Glutathione Participation in the Prevention of Cardiovascular Diseases

**DOI:** 10.3390/antiox10081220

**Published:** 2021-07-29

**Authors:** Deyamira Matuz-Mares, Héctor Riveros-Rosas, María Magdalena Vilchis-Landeros, Héctor Vázquez-Meza

**Affiliations:** Departamento de Bioquímica, Facultad de Medicina, Universidad Nacional Autónoma de México, Avenida Universidad 3000, Cd. Universitaria, Coyoacán, Ciudad de México 04510, Mexico; deya@bq.unam.mx (D.M.-M.); hriveros@unam.mx (H.R.-R.)

**Keywords:** cardiovascular diseases, glutathione, reactive oxygen species, reactive nitrogen species, oxidative stress

## Abstract

Cardiovascular diseases (CVD) (such as occlusion of the coronary arteries, hypertensive heart diseases and strokes) are diseases that generate thousands of patients with a high mortality rate worldwide. Many of these cardiovascular pathologies, during their development, generate a state of oxidative stress that leads to a deterioration in the patient’s conditions associated with the generation of reactive oxygen species (ROS) and reactive nitrogen species (RNS). Within these reactive species we find superoxide anion (O_2_^•–^), hydroxyl radical (^•^OH), nitric oxide (NO^•^), as well as other species of non-free radicals such as hydrogen peroxide (H_2_O_2_), hypochlorous acid (HClO) and peroxynitrite (ONOO^–^). A molecule that actively participates in counteracting the oxidizing effect of reactive species is reduced glutathione (GSH), a tripeptide that is present in all tissues and that its synthesis and/or regeneration is very important to be able to respond to the increase in oxidizing agents. In this review, we will address the role of glutathione, its synthesis in both the heart and the liver, and its importance in preventing or reducing deleterious ROS effects in cardiovascular diseases.

## 1. Introduction

Cardiovascular diseases, as occlusion of the coronary arteries, hypertensive heart diseases and strokes, arterial hypertension, myocardial infarction, cerebrovascular disease, heart failure, rheumatic heart disease, congenital heart disease and cardiomyopathies generate thousands of patients with a high mortality and morbidity rates worldwide [1,2,3,4]. The appearance of these diseases increases with the aging of the population [5,6]. In addition, these diseases are complicated by some comorbidity that patients present (overweight, obesity, diabetes mellitus, etc.). The installation and development of these diseases are closely linked to metabolic changes that generate a state of oxidative stress, due to the excessive production of reactive oxygen species (ROS) and reactive nitrogen species (RNS) [7,8]. It has been shown that the deficiency of antioxidant molecules in elderly humans is due to a significant reduction in their synthesis. A diet enriched with cysteine and glycine, the precursors of glutathione, fully restores cellular glutathione synthesis and GSH concentration, reduces oxidative stress levels and, thus prevents damages associated with oxidative stress and aging [9]. In the case of cardiovascular pathologies, the oxidative stress state leads to a deterioration in the patient’s conditions associated with the generation of ROS and RNS. The superoxide anion (O_2_^•–^), the hydroxyl radical (^•^OH), nitric oxide (NO^•^), and other non-free radical species such as hydrogen peroxide (H_2_O_2_), hypochlorous acid (HClO), and peroxynitrite (ONOO^–^) are all found within these reactive species [10]. NADPH oxidase (NOX), lipoxygenase, cyclooxygenase (COX), xanthine oxidase (XO), uncoupled nitric oxide synthases (NOS), cytochrome P450, and mitochondrial respiration are the most common enzymatic causes of ROS in CVD [11]. A molecule that actively participates in counteracting the oxidizing effect of reactive species is reduced glutathione (GSH). In the present review, we describe the role that glutathione plays in preventing an increase in ROS and RNS responsible for the development of cardiovascular diseases. Specifically, the following aspects are described: (i) description of ROS and RNS and antioxidant defenses, (ii) the importance of reduced glutathione (GSH) as an antioxidant agent, (iii) the participation of GSH in the prevention of cardiovascular diseases, (iv) the relationship between the production of reactive species and the development of cardiovascular diseases. Since several cardiovascular diseases are closely related to an increase in reactive oxygen and nitrogen species and, GSH is directly involved in reducing the harmful effects of both the disease itself and the oxidative effects of ROS and RNS, it will allow to obtain insights to design therapeutic strategies that will help restore normal physiological conditions.

## 2. ROS Excess and Antioxidant Mechanisms

As already mentioned, oxygen is essential for life, but its intermediaries can be a source of disease, through an uncontrolled production of ROS and more specifically of free radicals (FR) that damage the structure of organelles and macromolecules (lipids, proteins, carbohydrates and nucleic acids) [12,13]. Oxidative stress is a cellular condition that occurs as a result of the physiological imbalance between the levels of antioxidants and oxidants (either free radicals or reactive species) in favor of oxidants [14,15,16]. It is also related to aging and to diseases related to aging [5,6,17]. In eukaryotic cells, mitochondria, the cytochrome P450 (CYP) enzyme system, peroxisomes, and NADPH oxidases (NOXs) are the main contributors to ROS generation [18,19,20,21,22]. The ROS produced in these reactions can alter the intrinsic properties of the membrane such as fluidity, ion transport, loss of enzymatic activity, protein synthesis, DNA damage; which ultimately results in cell death [23,24].

Any alteration in cellular homeostasis leads to increased production of these FR, well above the detoxification capacity of local tissues [25,26,27]. In this process, more free radicals are often created, causing oxidative damage, which has been implicated as the cause of many diseases, such as cardiovascular diseases, neurodegenerative diseases, arthritis, diabetes, obesity, osteoporosis, metabolic syndrome, cancer, strokes, atherosclerosis, chronic inflammation and also has an impact on the aging process of the body [6,14,17,23,24,26,28,29]. External factors such as pollution, sunlight, smoking, unhealthy diets or conditions of nutrient deprivation and intense physical activity, also trigger the production of FR [14,20,21,29].

To prevent the damage caused to cells by oxidative stress, the body has a series of compounds called antioxidants that are characterized by preventing or delaying the oxidation of various biomolecules; therefore, oxidative toxicity is lower and with less cell damage. In addition, they promote the conversion of free radicals into inactive derivatives [6,14,23,26,30]. Antioxidant substances have been classified into two main systems, the enzymatic system and the non-enzymatic system; which can act both in the intracellular and extracellular space [14,19]. The first defense system corresponding to enzymes is based on an enzymatic defense complex that can include superoxide dismutase, catalase, glutathione peroxidase, thioredoxin reductase, and glutathione reductase [6,14,19]. The second, non-enzymatic antioxidant system is a parallel system to the first and is especially useful when the enzymatic system becomes saturated; it consists of a set of compounds that are mainly obtained from the diet [14,15,31,32]. This group of antioxidants is often referred to as radical scavenger as they dispose of FR by inhibiting the start of the oxidation chain and break chain propagation reactions, either by reducing or eliminating FR by donating one electron to them, without becoming free radicals or poorly reactive free radicals, with less damaging effects [16,17]. Other antioxidants in this category easily neutralize and render these ‘new radicals’ absolutely harmless. Some non-enzymatic antioxidants in cells are lipoic acid, glutathione, bilirubin, ubiquinones, bioflavonoids, vitamins C, E, A, and carotenoids [14,15,19,23,32,33,34]; while the minerals selenium, copper, zinc and magnesium are part of the molecular structure of some of the antioxidant enzymes [6,32,33,34,35,36].

### 2.1. Glutathione, a Key Intracellular Antioxidant

GSH is a tripeptide consisting of amino acids L-glutamate, L-cysteine, and L- glycine, having a molecular weight of 307.4 g mol^−1^. It is the most abundant non-protein thiol in cells; where it is present in intracellular concentration from 1 to 15 mM [37,38,39,40]. In contrast, the extracellular concentrations of this thiol are usually lower [41]. It may be present in the form of reduced thiol (GSH), as well as oxidized disulfide (GSSG); although under normal conditions almost 99% of cellular glutathione is in the form of reduced thiol [38,42]; about 1–2% glutathione in cells is in oxidized form and increases only under conditions of oxidative stress [43]. In cells, GSH performs a variety of functions that contribute to maintain cellular homeostasis [44]; however, the most important is its intracellular antioxidant activity, where glutathione is the most abundant cellular antioxidant [37,44,45].

### 2.2. Determination of Glutathione in Biological Samples

There are several problems for the determination of glutathione in biological samples, but the main one is the preservation and proper handling of the samples, since improper handling of it facilitates the artificial oxidation of the sulfhydryl group (-SH) of GSH, which acts as an electron donor. Additionally, in spite that the first determinations of GSH occurs more than fifty years ago, no broad agreement has yet been reached with relation to the most suitable preanalytical and analytical methods for the determination of glutathione in different types of samples. In consequence, a broad variation of the measured concentrations of GSH/GSSG by different laboratories has been reported. The main problem in many published papers is the overestimation of GSSG levels, since frequently an adequate treatment is not given to the samples during its collection and processing to avoid the oxidation of the GSH present. Consequently, this causes disagreement with the interpretation and comparison of the data obtained. Excellent reviews discussing extensively this problem have been published previously (e.g., [46]). In any case, this point is very important, because within cells the pool of GSH is 300 to 800 times greater than that of GSSG, therefore, even minimal artifactual or artificial oxidation of GSH can induce a dramatic increase in GSSG, without reflecting a true alteration in the cellular redox state, but even more so without indicating how this redox state can be affected by the health condition or disease that the patient presents.

### 2.3. Cellular Glutathione Synthesis

Glutathione synthesis takes place in the cytosol and is coupled with amino acid transport through the cellular membrane. It requires the consecutive activity of the enzymes gamma-glutamylcysteine synthetase (GCS) and glutathione synthetase (GSH synthetase). Both enzymatic steps require ATP as the energy source, and gamma-glutamylcysteine as the intermediary product [40,47,48], being the unusual gamma bond between glutamate and cysteine responsible for its cellular stability and resistance to degradation by intracellular peptidases; as a result, only the enzyme gamma-glutamyl transpeptidase, which is present on the bilayer lipid membrane’s exterior surface, can hydrolyze it [37,43]. This enzyme is important in glutathione metabolism, since, by breaking the gamma-glutamyl moiety of GSH, GSSG or GSH conjugates, the free glutamate is rapidly transferred to another external amino acid that originates a gamma-glutamyl-amino acid and the dipeptide cysteine-glycine [49]. The gamma-glutamyl amino acid is carried into the cell again where the amino acid is separated to form 5-oxoproline (the cyclic form of glutamate), which is then transformed into glutamate by the enzyme 5-oxoprolinase, and then used as such for GSH synthesis [43,47]. The dipeptide cysteine-glycine is degraded by the enzyme cysteine-glycine dipeptidase found on the cell membrane [50]. Both amino acids are reincorporated into the cell cytosol, where the released cysteine is mainly used in the synthesis of GSH, but another part is used in protein synthesis, or it is transformed into sulfate and taurine depending on the needs of the cell [38,51]. The active group of glutathione is the thiol (-SH) group found in cysteine [52].

### 2.4. Regulation of Glutathione Levels Is Modulated by a Diversity of Factors

**Figure 1:**
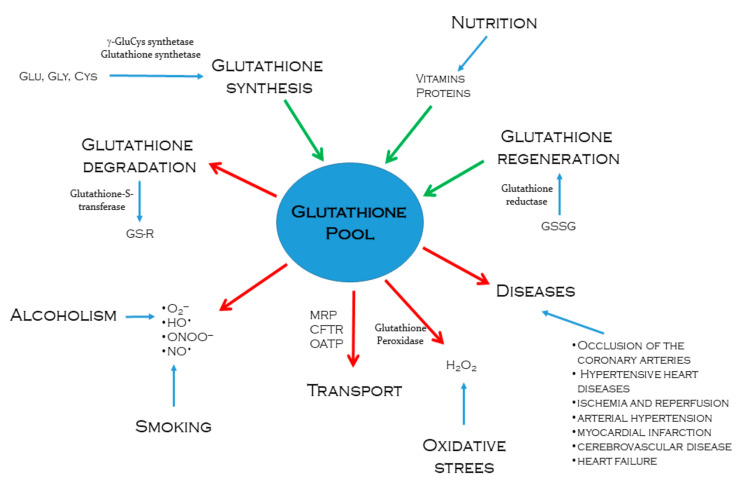
Processes that regulate intracellular glutathione pool. Green arrows indicate glutathione synthesis or regeneration through different metabolic pathways. Enzymes such as γ-glutamylcysteine synthetase, glutathione synthetase, and glutathione reductase are actively involved. The red arrows indicate the ways in which glutathione is used to neutralize and eliminate drugs or toxic metabolites to the body, facing oxidative stress caused by alcoholism and smoking. Intracellular glutathione also decreases as it is transported from the tissues that synthesize it in significant quantities to tissues that require greater antioxidant power.

The intracellular content of reduced glutathione is regulated according to its consumption and synthesis; it diminishes when used in conjugation reactions with xenobiotics through the glutathione S-transferase pathway or by forming GSSG due to its reactivity with free radicals and ROS [47]. Glutathione conjugates are exported and excreted into the bile, provoking an irreversible loss of the cysteine residue of glutathione, a limiting step for its synthesis; hence, this detoxification pathway consumes intracellular glutathione in an irreversible way [38]. However, GSSG may be reduced to regenerate glutathione by NADPH-dependent glutathione reductase enzyme; although the recovery of glutathione contents is mainly due to de novo synthesis [47]. According to nutritional conditions, the levels of amino acids may vary, being cysteine, glutamate, and glycine the most important for glutathione synthesis; nevertheless, since even in normal nutritional conditions, cysteine is the amino acid present in the lowest concentration, its availability is as a rule the limiting factor in GSH synthesis [53]. Modulators of glutathione pool are summarized in Figure 1.

The methods used to increase the intracellular levels of glutathione that have shown efficacy are the administration of compounds that favor cysteine availability [45]. Some of the most widely used are:

- Methionine as a precursor of cysteine. In cells, methionine is transformed into cysteine through the transsulfuration pathway, by the action of the enzyme cystathionine β-synthase and cystathionine γ-lyase or cystathionase [38,54,55,56]. Disruption of the transsulfuration pathway contributes to the pathology of several conditions, such as vascular dysfunction [56].
- N-acetylcysteine (NAC) functions as a precursor of the amino acid L-cysteine, fostering the intracellular production of GSH [45,57]. It has been shown that many types of cells can trap NAC, hydrolyze it and change it into L-cysteine, which is then incorporated into the cycle of gamma glutamyl, stimulating glutathione synthesis [58].
- S-Adenosyl-L-Methionine (SAM), metabolite involved in the transsulfuration pathway. First, the methyl group of the molecule must be released, forming S-adenosyl-homocysteine, which is transformed into homocysteine, and then into cysteine [43,56,59].
- L-2-oxothiazolidine-4-carboxylate (OTC), an analogous of 5-oxoproline (which is the cyclic form of glutamate), increases the cellular levels of cysteine and glutathione [60,61].
- Alpha-lipoic acid (α-AL) is considered the “universal antioxidant” because it is an amphipathic molecule that may act in both aqueous and hydrophobic environments. The organism, using NADH or NADPH, usually transforms it into dihydrolipoic acid (DHLA), reduced form of α-AL, which has an important antioxidant effect and can reduce GSSG directly into GSH increasing the intracellular levels of reduced glutathione [62,63]. DHLA may also be released into the extracellular space where it reduces cysteine, which can be taken and transported into the cell and used in the synthesis of glutathione [63,64,65].
- The monoester of GSH, which can be transported into many tissues (lung, kidney, heart, liver) also increase glutathione levels [57,66].
- The diester of GSH is effectively transported into cells, hydrolyzed to GSH, thus increasing glutathione levels. It is four times more effective than GSH monoester [61].

GSH cannot be administered directly because two pharmaceutical problems have been observed: poor bioavailability with oral administration, and a short half-life (2 min) with intravenous administration [54]. Thus, administration of GSH could not be the best solution because intestinal and hepatic gamma-glutamyl transferase (GGT) metabolizes GSH and decreases the bioavailability of administered GSH [67,68]. However, healthy participants were tested after receiving pure GSH in the form of an orobuccal pill with a fast-slow release. It was discovered that increased GSH levels in the blood could be due to GSH absorption through the oral mucosa in this assay. Other researchers evaluated the levels of GSH and other oxidative stress markers in the blood of metabolic syndrome patients after receiving various forms of GSH (oral and sublingual) and NAC. They showed that administration of sublingual GSH compared to oral GSH increases the level of GSH and the GSH/GSSG ratio. These findings suggest that taking the sublingual form of GSH may be a viable therapy option to reduce oxidative stress and prevent oxidative stress-related illnesses [69,70].

### 2.5. Glutathione in Liver and Heart

In the liver, the role of glutathione (GSH) as an antioxidant is especially relevant because it is its main site of synthesis, storage, and export [43,49]. The importance of GSH in the liver lies on the central role that this organ has as responsible for the oxidation and elimination of substances such as ethyl alcohol and other toxic products that induce oxidative stress; therefore, the liver requires the presence of antioxidant agents which prevent or reduce this stress, either by trapping, metabolizing or transforming molecules into agents less toxic than ROS [71].

GSH plays a vital role in the protection against oxidative stress, since it traps ROS [72,73]. GSH may react with different free radicals such as hydroxyl radical, hypochlorous acid, superoxide, peroxynitrite radical, and reduces hydrogen peroxide, thus being the first cellular defense line against oxygen reactive species [26,43]. The second defense line are the antioxidant enzymes which glutathione uses as cofactor [48,74].

GSH plays an important role in the cardiovascular system because it is an important antioxidant that restores intracellular redox equilibrium and prevents the inactivation of nitric oxide produced by the endothelium, leading to aberrant vasomotor reactivity in individuals with coronary spastic angina [75,76]. CVD are closely associated with redox imbalances, such as hypertension and atherosclerosis. Increases in the accumulation of reactive oxygen species are related to polymorphisms in antioxidant enzymes such as glutathione peroxidases and glutathione S-transferases, which are linked to an increased risk of vascular disease. Glutathione peroxidase polymorphisms seem to increase the risk of developing coronary artery disease, stroke, and cerebral thrombosis. In smokers, the increased risk of coronary heart disease is related to glutathione S-transferase polymorphisms [77].

### 2.6. Plasma Glutathione

Multiple processes control the plasma GSH/GSSG redox state, including GSH synthesis from its constitutive amino acids, cyclic oxidation and reduction involving GSH peroxidase and GSSG reductase, GSH transport into the plasma, and GSH and GSSG breakdown by γ-glutamyltranspeptidase [49,78,79]. GSH is present in all mammalian cells in a constant state of metabolic recirculation (synthesis, degradation, and irreversible loss of GSH), its half-life is 4 days in human erythrocytes, 2 to 4 h in the cytosol of rat hepatic cells and 30 h in the mitochondrial lumen [40,80]. Many different conditions affect the contents of intracellular GSH, some of them are the presence of heavy metals, high glucose concentrations, protein malnutrition, thermal shock, exposure to reactive oxygen and nitrogen species including, H_2_O_2_ and nitric oxide, ozone exposure, ionizing radiation, and cigarette smoke [37,40,61,73,81].

### 2.7. Other Functions of Glutathione

Some of the other functions performed by glutathione are: T-lymphocyte activation; hence, it plays an important role in the immune response [45,49,82]. Glutathione also participates in gene expression regulation processes, signal transduction, DNA and protein synthesis regulation, as well as proteolysis [82,83], cellular proliferation including lymphocytes and intestinal epithelium cells [37,73,84], apoptotic processes regulation, cytosine production [38,49,85], protein glutathiolation [49,74], regulation of mitochondrial integrity and functioning [39,73,86]; it has a vital role in spermatogenesis and spermatozoon maturation [73,87], it inhibits viral infections (e.g., influenza) [88], it has an important role in the pathogenesis of different diseases such as cancer, inflammation, protein deficiency, Alzheimer’s disease, Parkinson’s disease, hepatic diseases, cystic fibrosis, HIV, heart attacks, hemorrhagic stroke, diabetes, pulmonary disease, gastritis, duodenal ulcer, pancreatic disease, kidney disease, rheumatoid arthritis (RA), autoimmune thyroiditis, muscular dystrophy, amyotrophic lateral sclerosis, preeclampsia, cataract, glaucoma, atherosclerosis, and asthma [37,51,52,73,85,89,90,91,92,93,94,95,96].

### 2.8. Glutathione Transport Is Performed by Three Different Protein Families

The first step in glutathione movement in the liver is its transport through the cell membrane to the extracellular space, in order to be used by extrahepatic organs and tissues [97]. Glutathione synthesis takes place in the cytosol [73], while its degradation only takes place in the extracellular space, particularly on cells that express the enzyme gamma-glutamyl transpeptidase, which is the only enzyme that can hydrolyze it [43]. Most transport epithelia, including the hepatic canaliculus and bile duct membranes, have this enzyme abundant on the apical surface [97]. Since gamma-glutamyl transpeptidase is the only enzyme capable of starting the catabolism of GSH, GSSG, and glutathione S-conjugates, it has been proposed that it is the enzyme responsible for the regulation of glutathione movement in mammal cells [97,98]. It is more relevant in the liver, the main site of synthesis, storage, and distribution of glutathione, as well as the amino acids that constitute it. Hence, the liver is really important in the regulation of organic homeostasis of such thiol [99]. Interestingly, glutathione efflux can be stimulated through β-adrenoceptors activation [100].

Considering that glutathione is involved in the elimination of harmful substances such as: alcohol, drugs, solvents, pesticides, heavy metals, and many other toxic xenobiotic products that induce a state of cellular stress [71], it is evident that glutathione homeostasis becomes more relevant, because cells require an antioxidant agent that either prevents or reduces this stress, whether by trapping, metabolizing or turning them into less toxic agents [71,101]. This is why the liver is an organ with a large amount of enzymes that participate both in the synthesis and in the formation of glutathione conjugates as products of the elimination of xenobiotics. It was believed that the hepatocytes also have the unique capacity of converting methionine into cysteine through the transsulfuration pathway [38,102]; however, more recent data supports the view that transsulfuration plays a physiological role in GSH production in the brain and in astrocytes in particular [83]. In fact, cystathionine γ-lyase, and the enzyme belonging to the transsulfuration pathway are expressed in many peripheral tissues, including the aorta and the heart [103,104]. This last pathway is relevant due to the role of glutathione as a means of storage and transport for cysteine, an important agent in extracellular reduction reactions, a critical substrate for protein synthesis, and a limiting precursor in taurine synthesis [102,105].

Many glutathione transport mechanisms are present in hepatic cells:

- The sinusoidal transport system, found on the basolateral membrane of hepatocytes, releases GSH into the blood [106].
- The system found on the canalicular membrane that transports GSSG and glutathione S-conjugates to the bile, and plays an important role in the hepatic detoxification of drugs, metals, and other reactive compounds, both endogenous and exogenous [106,107].

The scanty information we have about glutathione transport and movement is mainly due to the theoretical and practical difficulties that have not allowed the functional and molecular description of glutathione transporters [97,108].

Recent studies have shown that three transporter families present in mammal cells participate in glutathione transport and movement, and are called: (i) Multidrug Resistance-associated Protein (MRP) Family, (ii) cystic fibrosis transmembrane conductance regulator (CFTR) family, and (iii) organic anion-transporting polypeptide (OATP) family [97,109].

MRP proteins belong to the family C of the ABC-transporter superfamily, which require ATP to perform GSH transport [110]. MRP discovered in cancerous cells as integral membrane proteins; it has been shown that they transport organic ions, mainly glutathione S-conjugates in an ATP-dependent way [109,111], GSSG is another transport substrate for them [112]; although these transporters have low affinity for GSH [97].

In the liver, the MRP transporters are mainly present on the epithelial cells of the bile duct, on the sinusoidal membrane, and on the basolateral membrane [109]; therefore, it is considered that the main function of MRPs is the transport into the bile duct, not into the blood stream, and that glutathione is required only by this transporter family as a co-transporter of other substrates for MRP (for instance, estrogens and sulfates) [113,114].

CFTR proteins belong to the C family of the ABC-transporter superfamily [110,111,113]. CFTR proteins are ion channels located on the apical surface of mucous membrane and submucous gland epithelial cells [115]. They are activated by cAMP and may be regulated by phosphorylation catalyzed by protein kinase A and C (PKA and PKC), and require the binding and hydrolysis of ATP to open the channel [100,116]. FTR regulates chlorine (Cl^–^), bicarbonate (HCO_3_^–^), sodium (Na^+^) and also facilitates GSH transmembrane transport [115,117].

Organic anion-transporting polypeptide (OATP) family function independently from ATP and sodium gradient [118], and is found on the basolateral membrane of the hepatocytes and on the apical membrane of intestine epithelial cells [119], using the following transport substrates: bile salts, organic anions, steroids, leukotrienes, and prostaglandins [120]. Members of this family have also been found in the placenta where they are thought to participate in steroid transport mechanism [121]. Just two members of OATP family (OATP-1 y OATP-2) accept GSH as substrate, releasing it into the blood stream; OATP-1 is homogeneously distributed throughout the hepatic acini, while OATP-2 is mainly expressed in perivenous hepatocytes [107,122]. Membrane potential is the force used by these transporters to transport GSH; therefore, depolarization and hyperpolarization of the membrane potential either diminishes or increases the release of GSH, respectively [97].

In mitochondria, a GSH intracellular transport system located on the inner mitochondrial membrane and that mediates the movement of GSH from the cytosol into the mitochondrial matrix was reported [106,107,123]. Therefore, GSH concentration in mitochondria is kept constant due to two transport systems: a high-affinity one, ATP-dependent, and a low-affinity one, ATP- and ADP-dependent [124,125]. Subsequent studies identified mitochondrial dicarboxylate carrier (DIC), and the 2-oxoglutarate carrier (OGC) as the protein transporters responsible for GSH influx into mitochondria [126,127]. However, a recent paper using vesicles from *Lactococcus lactis* overexpressing DIC and OGC questioned this conclusion, since they could not measure GSH transport, even under glutathione excess conditions [128]. Thus, the identity of the mitochondrial glutathione transporters is uncertain.

## 3. Role of Oxidative Stress in the Generation of Cardiovascular Diseases

An imbalance in the redox homeostasis could cause cardiovascular complications. ROS are produced by all vascular layers, and they act as signaling molecules that regulate several functions such as vascular smooth muscle cell contraction, relaxation, and growth [129]. In contrast, excessive or sustained increase in ROS generation plays an essential role in endothelial dysfunction (ED) and CVD development. Some cardiovascular risk factors are associated with either increased production of ROS or decreased plasma GSH levels [130]. In both cases, cell damages are due to direct oxidizing effects on proteins, lipids, and DNA [10].

Changes in GSH concentration or oxidation status have been linked to the development and progression of CVD [131]. Many studies exhibit the effect of different proteins that modulates the role of GSH in CVD. For example, heat shock proteins 27 (Hsp27) and 25 (Hsp25) in humans and mice (L929 fibroblasts) give protection against H_2_O_2_ by raising levels of reduced GSH [132]. Furthermore, it has been found that degradation of transcription factors such as nuclear erythroid 2 related factor (Nrf2) decreases expression of many antioxidant enzymes. Also, NOX4 increases concentrations of GSH by activating Nrf2-regulated pathway [133,134]. The following paragraphs describe some cardiovascular diseases generated by oxidative stress:

### 3.1. Endothelial Dysfunction (ED)

An imbalance of nitric oxide (NO^•^) and ROS may promote ED, leading to cardiovascular complications. The first change seen is endothelial activation, which is characterized by an aberrant pro-inflammatory and pro-thrombotic phenotype of blood vessel endothelial cells. ED is caused by a decrease in NO^•^ bioavailability, a decrease in vascular tone, and other phenotypic alterations in the endothelium [135]. NO^•^ is a molecule with vasodilatory properties and plays a significant role in vascular homeostasis. High levels of superoxide may react with NO^•^ and generate peroxynitrite, which is a harmful free radical [136]. On the other hand, under certain conditions such as low availability of substrate or cofactors, endothelial nitric oxide synthase (eNOS) can produce superoxide instead of NO^•^ in a condition known as uncoupling. Also, ROS activate the MAPK pathway and inhibit eNOS mRNA expression and eNOS activity [137]. All these circumstances decrease the amount of NO^•^ and cause the loss of endothelial function.

On the other hand, it has been reported that GSH is essential for endothelial function and anti-fibrotic response in the kidney. The decrease in GSH in the endothelium induces ROS levels, decreases the activity of eNOS, and increases organ fibrosis [138]. Also, ED caused by cigarette smoke is protected by glutathione-S-transferase P (GSTP) [139]. Recently, it has also been showed that enhancing antioxidant pathways can be helpful in reduce ED in uremia and a possible target for lowering the cardiovascular risk of kidney disease [140].

### 3.2. Hypertension and Diabetes Mellitus (DM)

ROS and oxidized low-density lipoproteins (ox-LDL) participate in the pathophysiology of hypertension. They may inactivate NO^•^, causing arteriolar vasoconstriction and elevation of peripheral hemodynamic resistance. Hypertensive patients produce excessive amounts of ROS concomitant with a decreased antioxidant capacity. Activation of the renin-angiotensin system is a significant mediator of NOX activation and ROS production in human hypertension. Angiotensin II (Ang II) regulates hypertension by the renin-angiotensin system and the stimulation of NOX in vascular walls. Endothelin-1 (ET-1) increases NOX activity in human endothelial cells. Platelet-derived growth factor (PDGF), transforming growth factor-beta (TGF-β), tumor necrosis factor-beta (TNF-β), and thrombin also activate NOX in vascular smooth muscle cells (VSMC). On the other hand, antihypertensive drugs, calcium channel blockers, and angiotensin receptor blockers decrease the expression of NOX subunits and their activity [136].

Furthermore, excessive oxidative stress appears to be key in type 2 diabetes mellitus (T2DM), and ROS/reactive nitrogen species (RNS) play important functional and dysfunctional roles at the cellular level, especially in tissues that affect T2DM, such as pancreatic islets, muscle, adipose tissue, and liver. Chronic hyperglycemia causes an increase in ROS/RNS production, which promotes the development of secondary diabetes problems. Overproduction of reactive oxygen species (ROS) disrupts cell function and causes cell death in tissues such as the kidney, peripheral nerves, and the circulatory system [10,141].

Several others studies indicate that people with CVD or with T2DM have decreased level of GSH in their blood [130]. In other studies, the level of GSSG and TGF-β was higher in diabetic patients. In this case, an increased level of proinflammatory cytokines and decreased expression of enzymes involved in GSH synthesis were observed [142]. Additionally, it has been seen that it is possible to decrease systolic and diastolic pressures by increasing GSH concentration and, thus decrease diabetes incidence. Also, it has been found that hypertensive patients have low concentration of GSH and high level of GSSG, and the antihypertensive therapy reduced oxidative stress [143]. Furthermore, GSH is decreased in symptomatic CVD patients [144]. All of these studies support the idea that decreases in GSH levels are related to the development of CVD.

### 3.3. Atherosclerosis

Free radicals are involved in the atherogenic process. Various isoforms of NOX (NOX1, NOX2, and NOX4), xanthine oxidases, and mitochondrial enzymes produce ROS at physiological levels. They can become pathologic due to excess production or failure of antioxidant mechanisms [145]. Hydroxyl radicals cause the peroxidation of polyunsaturated fatty acids within LDL molecules. NOX is activated more by ox-LDL than by native-LDL, and reduces eNOS activity [136]. The modified lipoprotein particles cause monocytes and T-lymphocytes to be recruited into the sub-endothelial region by increasing the expression of cell adhesion molecules in endothelial cells. Ox-LDL also stimulates the release of monocyte-derived tumoral necrosis factor alpha (TNF-α) and interleukin 1 beta (IL-1β), leading to smooth muscle cell proliferation. Plaques and fibrosis are caused by extracellular matrix protein generated by these cells. Prostacyclin production is inhibited by lipid peroxides, which might lead to platelet adhesion and aggregation. Smooth muscle cells proliferate and migrate to the intima when platelets release growth factors. Furthermore, this may result in thrombosis [146].

In mice with atherosclerosis or apolipoprotein E-deficient mice, treatment with liposomes containing GSH reduced serum susceptibility of lipoproteins to 2,2′-azobis(2-amidinopropane) dihydrochloride (AAPH) oxidation [147]. Another study showed that mice on a high saturated fat diet treated with N-acetylcysteine (NAC) could increase GSH levels, and reduced concentrations of cholesterol [148]. In humans, the effect of antioxidants in patients with atherosclerosis has also been studied. Results showed that administration of NAC increased levels of GSH and reduced levels of endothelial adhesion molecules and could prevent vascular damage in patients with diabetes [149]. Also, it has been shown that GSH reduced peripheral vascular endothelium-dependent vasodilation, in patients with atherosclerosis by increasing NO^•^ activity [150]. These results showed how glutathione has antioxidant and antiatherogenic properties and may lead to remission of atherosclerosis.

### 3.4. Cardiac Hypertrophy

Many stimuli as Ang II, ET-1, tumor necrosis factor alpha (TNF-α) could induce the hypertrophy of myocytes by ROS action [10,151]. ROS active many signaling kinases (e.g., mitogen activated protein kinases), transcription factors (e.g., nuclear factor kB [NF-kB] and nuclear factor of activated T-cells [NFAT]), molecules that participate in calcium mobilization (e.g., ryanodine receptors [RyR2], SERCA 2A, calcium/calmodulin-dependent protein kinase II [CaMKII]), antioxidant proteins and others molecules involved in cardiac hypertrophy [151,152]. Other data supporting ROS participation in this process are that N-acetylcysteine restores total myocardial glutathione, lowers oxidative stress, and reduces myocyte hypertrophy and fibrosis in aortic stenosis rats [153]. In addition, drugs such as trimetazidine (TZM) decrease cardiac hypertrophy by increasing the level of antioxidant enzymes such as catalase, superoxide dismutase and GSH peroxidase [154].

### 3.5. Ischemia-Reperfusion Injury

When the blood flow is disrupted (ischemia) and then re-established (reperfusion), Ischemia-Reperfusion (I/R) damage occurs. During ischemia, ROS levels are modest, but when oxygen supply is restored during reperfusion, high levels of ROS and substantial damage to cardiac cells are produced. Myocyte death in I/R is linked to ROS-induced mitochondrial depolarization during ischemia. The opening of the mitochondrial permeability transition pore (mPTP) may be responsible for the reduction in membrane potential [155]. In addition, it has been suggested that the disruption of the mitochondrial inner membrane by ROS-induced lipid peroxidation contributes to ischemia injury. During ischemia, a large increase in the antioxidant defense system, such as glutathione augmentation, has been seen in an attempt to deal with oxidative stress [156,157]. Moreover, N-acetylcysteine, angiotensin-converting enzyme inhibitors, catalase, mannitol, superoxide dismutase, and vitamin E have been shown to have antioxidant properties that help to prevent I-R injury [158].

### 3.6. Heart Failure

During the transition from compensatory hypertrophy to cardiac failure, oxidative stress appears to play a critical role. Superoxide is produced by a variety of enzyme systems, and mitochondria appear to be an important source of myocardial ROS in the failing heart [136]. Failing cardiomyocytes have reduced Ca^2+^ levels in the sarcoplasmic reticulum (SR). ROS directly increase the sensitivity of ryanodine receptors (RyR) in heart failure by oxidizing cysteine thiols. This effect increases Ca^2+^ release by SR [159]. Vitamin E, hydroxyanisole and catalase prevented hypertrophy induced with TNF-α and Ang II in cardiac myocytes [160]. Furthermore, increased peroxynitrite may also contribute to cytokine-induced cardiac contractile failure by inactivating sarcoplasmic Ca^2+^-ATPase and disrupting Ca^2+^ homeostasis [161]. Likewise, the activity of XO and NOX increases in the failing heart. These results indicate that oxidative stress participate in the pathophysiological cardiac dysfunction in heart failure [136].

Moreover, antioxidant enzymes participation has been studied in the generation of CVD and other diseases. For example, some glutathione S-transferase (GST) polymorphisms increase risk of myocardial infarction (MI) in cardiac surgery. Additionally, decreased glutathione peroxidase (GPx-1) activity has been found to increase the risks of stroke and coronary heart disease. Thus, the measurement of the level of GPx-1 and the GST polymorphism determination could be used as predictive values for CVD. Furthermore, GPx-1 can be considered as an enzyme that can prevent the development of ED and atherosclerosis in other diseases such as T2DM [162].

## 4. Discussion and Conclusions

Many diseases are due to an imbalance between oxidant production rate (free radicals or reactive species), and the activity of cellular antioxidant systems. Glutathione plays a central role as antioxidant defense, as well as in the regulation of pathways involved in cellular homeostasis, not only as a detoxifier of endogenous and exogenous compounds, but also through its participation in processes related to cell proliferation, apoptosis, gene expression, regulation of the immune system, and metabolism of cell compounds, among others. Disturbances in GSH homeostasis are related to the etiology and/or progression of a variety of diseases besides the CVD listed in this work, such as cancer [163,164] diabetes [165,166,167], cystic fibrosis [168,169], Parkinson’s disease [170,171,172], Alzheimer’s disease [173,174], as well as inflammatory [175], immune [176,177], metabolic [178], and neurodegenerative diseases [179,180], among others. All these pathologies share a reduction in the glutathione pool that usually concurs with an increase in the production of ROS and RNS. Even GSH depletion has been associated with higher rates of serious illness and death in patients with coronavirus disease (COVID-19) [181,182,183].

Another aspect to highlight is the importance of the metabolism, cell distribution, and transport of said thiol. Glutathione transporters are of utmost importance because they minimize the fluctuations in the GSH concentrations, as well as in the redox state of glutathione, in the different cellular compartments, while their synthesis, degradation and recycling act in a coordinated form.

**Figure 2:**
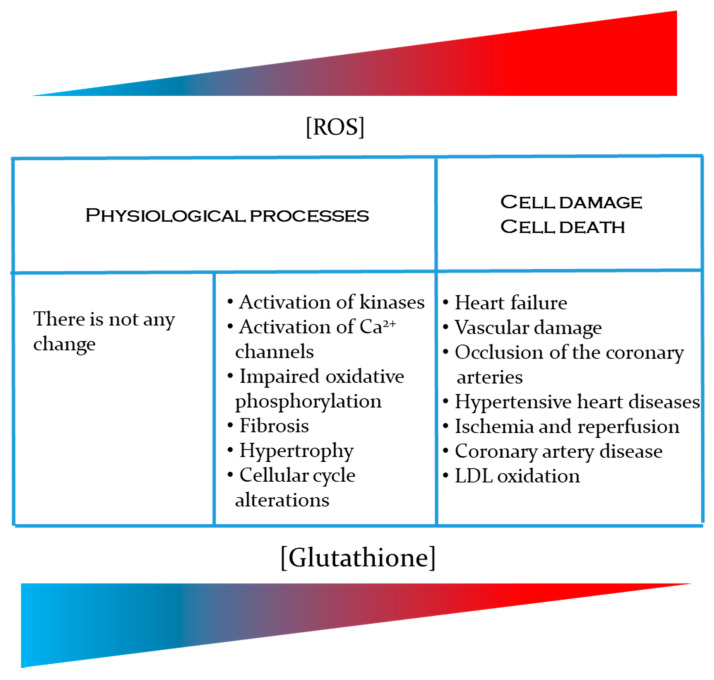
Importance of GSH in the prevention of cardiovascular diseases related to an increase in the production of reactive oxygen species [ROS] and reactive nitrogen species [RNS].

On the other hand, it is well-known that glutathione deficiency contributes to the oxidative stress, and has an important role in aging, as well as in the pathogenesis of different cardiovascular diseases. All this makes the study of this small tripeptide even more interesting. In summary, the information in this paper covers the journey from the basic physiological mechanisms for glutathione synthesis and regulation through to how glutathione ameliorates free radical imbalances at the organ system level (Figure 2). However, it is important to note that at population-level, the importance of GSH in the development of CVD has been demonstrated. For example, a cohort study of CVD in the town of Hisayama showed that people with a reduced plasma level of GSH are at increased risk for CVD, especially for cerebral small vessel disease [130]. In the same way, the impact of diet on GSH levels [184], as well as in the development and progression of CVD in different population or groups of patients has also been demonstrated. Bettermann [185] for example, performed a cross-sectional study with 685 volunteers, showing that adherence to the Mediterranean diet was positively associated with higher plasma GSH levels. Similarly, Yubero-Serrano et al. [186] in a controlled clinical trial with 1002 coronary heart disease patients concluded that the Mediterranean diet modulates better endothelial functions, even more than low-fat diets unlike western high-glucose diets that are associated with a higher risk of CVD (e.g., [187]. If glucose is available in excess, aldose reductase convert glucose into sorbitol through the polyol pathway [188]. Since this pathway use NADPH as electron donor, this compromises the availability of this coenzyme to regenerate reduced glutathione, exacerbating the oxidative stress [189].

Although there is a great amount of information about glutathione, it is evident that there is still a lot to discover about its function in cellular regulation. In the last years, approaches to design future therapies consider novel aspects as, for example, heritability of GSH levels in different organs/tissues [190], regulation of epigenetic mechanisms as response to variations of glutathione level or GSH/GSSG ration [191]; redox signaling through the formation of mixed disulfides stimulated by hormones [192] or even, testing in animal models, a mitochondrial transplantation therapy that could increase ATP supply and reduce ROS damages [193]. Indeed, Cowan et al. [194] successfully reported intracoronary exogenous mitochondrial delivery into an ischemic heart for cardioprotection. Therefore, the study of glutathione remains an important and extensive research field which requires further examination.
